# Destruction of the Entire Palate Successfully Treated by Mechanical Means

**Published:** 1857-01

**Authors:** 


### ARTICLE XIV.

***Destruction of the Entire Palate successfully treated by Mechanical Means.*** Mr. Sercombe communicated the following interesting case to the ***Royal Medical and Chirurgical Society***, London.

THE author stated that he had no intention to refer to the form or character of the disease which produced the consequences related in his paper, but that his sole object was to establish the fact, that it is possible to restore, by mechanical contrivance, one or more portions of the face which may have been destroyed by disease or injury. Mr. Sercombe's patient had been driven from a post of public duty which he had adorned, after fourteen years of terrible suffering, fearfully disfigured, and rendered quite unintelligible to those around him. The progress of the disease and present condition of the patient were thus described:—The soft parts in the fauces were attacked with the most virulent ulcerations, and sloughed away in masses. The upper front teeth became black, and several were removed; the gums ulcerated, and the alveolar processes exfoliated. In this way the upper incisors, canines, and bicuspids were lost; three molars remaining on one side, and two on the other only. The ulceration next crept forwards to the face; the nose was attacked, and partially destroyed, the lips completely so; indeed the whole of the face below a straight line drawn from one meatus auditorius externus over the lower margin of the nasal bones to the other meatus, was subjected to the same destructive process, and was intersected in every direction with cicatrices. The intermaxillary and palate bones exfoliated in large pieces, accompanied with serious hemorrhage from the palatine, and other smaller arteries. The vomer and central lamella of the ethmoid also disappeared. Considerable portions of the inferior maxillary bone exfoliated, and all the teeth, save one canine and two incisors, were lost. External examination of the face showed partial destruction of the alæ nasi, particularly of the right side; complete loss of both lips, as the band stretching across in the place of the upper lip scarcely deserved that name, being a firm, unyielding mass of abnormal tissue. The cavity of the mouth was thus at all times exposed through an irregularly oblong opening, on the lower border of which stood the three teeth of the lower jaw. The effect of the formation of the cicatrices which covered the face in all directions had been to contract it at least one inch and a half, measuring in a straight line from the margin of the hair on the forehead to the lower border of the inferior maxilla; added to this, the greater part of the hole leading into the mouth was placed very much to the right of the mesial line, and the rigid condition of the cicatrices prevented the lower jaw being depressed more than the eighth of an inch. The paper was well illustrated by casts and models both of the face before the commencement, and after the arrest of the disease, and of all the appliances so successfully contrived by the author. Such was the most unpromising state of things when Mr. Sercombe was introduced to the patient. His first intention and utmost hope, was to contrive an obturator which should render the wearer intelligible to his own family and friends. The first step was to remove the three teeth, already alluded to, as previous to that being done, a single finger could not be introduced into the mouth. Still much more space was required to work in, and an attempt was made to enlarge the opening towards the left ear by an incision of from an inch and a half to two inches. This proved, however, unsuccessful, in consequence of the contraction of the wound during cicatrization. It became clear that the ordinary mode of moulding the whole, or even half the jaw, could not be employed in this case, for it was impossible to obtain a model in one piece. After ingeniously overcoming this difficulty by the introduction of separate portions of softened wax, and forming a model of the whole combined in plaster of Paris, a gold plate was fitted to each; but much difficulty was experienced, and great patience and ingenuity exhibited before the true relative positions of the two side plates, and thus the size and width of the centre plate, or palatine arch, could be determined. It was eventually obtained by the use of a plate of Britannia metal, which could be easily moulded with the finger. In this way the working model was obtained, upon which two lateral and a centre gold plate were formed. They are introduced into the mouth separately, and firmly joined together when *in situ;* the central one to that on one side by a grooved border, to that on the other by a hinge into which a fine gold pin fits, one division of the hinge being on the lateral piece, and two others on the central. The tongue assists in placing the parts *in situ*, and it is effected in less than two minutes. Although by this means the voice was at once restored, articulation was still imperfect, and it was determined to attempt to supply an artificial soft palate, and after numerous fruitless experiments, one was contrived of vulcanized india-rubber, which was as successful as could be expected in the absence of lips. To the restoration of the lips, Mr. Sercombe, therefore, next directed his energies, and in the first place again resorted to vulcanized india-rubber, thin gold bands being secured to each lip, by which the wire attached the upper to the centre plate, and the lower to a plate fitted to the lower jaw for the express purpose of carrying this lip, by which arrangement they could be placed *in situ* and removed at pleasure. With the apparatus thus completed, the power of distinct articulation was within a week so far restored that the patient returned to the country to resume duties involving public speaking, from which he had been for so many years, entirely excluded. Some modifications have been rendered advisable, because, during the last twelve months, the material covering the chin has become softer, fuller, and more movable, presenting grounds for hope that ultimately, a useful natural lip will be formed; in order not to interfere with this progress, the lower artificial lip has been dispensed with. Ivory has been substituted (without any disadvantage to the voice or articulation) for india-rubber in the upper lip; the latter material was found to soften and decompose at the edge, and the paint with which it was colored, to imitate the skin, peeled off. The ivory has also these advantages, that it can be more firmly fixed, and teeth showing below the lip, may be attached to it. The apparatus has been worn for nine months, and in a letter recently received, the patient stated his voice to be as good as he could possibly expect,—almost as good as he could wish. This, however, was not the only advantage gained; the apparatus has been found to effectually prevent the passage of fluids from the oral to the nasal cavity during deglutition. The sense of taste, which was lost, has been restored; that of hearing also, which was completely destroyed in the right ear, and nearly so in the left, has been fully restored to the latter, although it remains unimproved in the former. The cause of deafness would seem to have been the extension, through the Eustachian tubes, of a dried and thickened condition of the mucous membrane of the pharynx, induced by constant direct exposure to the atmosphere at the ordinary temperature. Mr. Sercombe concluded his paper with these three remarks: First, that it matters little how long or wide the cleft may be, an obturator can be as easily adapted to the largest as to the smallest opening, and, therefore, the possibility of an operation for cleft palate being followed by sloughing, which would enlarge the primary opening, ought not to present any grave objection to its being performed; and, therefore, in all cases where there is no contra-indication, the chance of a successful issue should be given. Second, in cases where the fissure is accidental and not congenital, it requires but that the parts lost be restored, and almost immediately the power of speech is regained. Third, that portions of the face, which may have been destroyed by accidental causes, may be restored by mechanical contrivance when beyond the reach of the surgeon.—*Lond. Lan.*

